# Physical fatigability and muscle pain in patients with Hashimoto thyroiditis

**DOI:** 10.1007/s00415-020-10394-5

**Published:** 2021-01-28

**Authors:** B. Jordan, O. Uer, T. Buchholz, A. Spens, S. Zierz

**Affiliations:** 1 Department of Neurology, University of Halle, Saale, Germany; 2 Department of Neurology, University of Heidelberg, Im Neuenheimer Feld 400, 69120 Heidelberg, Germany; 3 Department of Internal Medicine II, University of Halle, Saale, Germany; 4 MVZ Stoffwechselmedizin Leipzig, Leipzig, Germany; 5 Heart Center, Department of Cardiac Surgery, University of Bonn, Bonn, Germany

**Keywords:** Hashimoto thyroiditis, Pain, Fatigue, Fatigability, Linear trend

## Abstract

**Introduction:**

Hashimoto thyroiditis (HT) may lead to muscle weakness due to hypothyroid dysfunction. However, clinical experience treating patients with HT suggests that neuromuscular symptoms may develop in these patients despite long-standing euthyroidism.

**Methods:**

In 24 euthyroid patients with HT and 25 healthy controls, physical fatigability was assessed using the arm movement test (AMT) and 6-min walk test (6MWT). Fatigability was based on calculation of linear trend (LT) reflecting dynamic performance within subsequent constant time intervals. Perception of physical fatigue and muscle pain was analyzed using fatigue (FSMC) and pain questionnaires. Obtained results were correlated with clinical, neurophysiological and lab findings.

**Results:**

HT patients showed a negative LT in 6MWT significantly differing from stable performance in controls. LT in AMT did not differ between HT and controls. FSMC scores and pain perception revealed significantly higher levels in HT patients than in controls. Physical FSMC score was primarily influenced by pain perception (standardized regression coefficient, beta = 0.633, *p* = 0.002). Neither pain score nor physical fatigue score showed a correlation with LT in 6MWT nor did mood, or anti-TPO antibody titer.

**Conclusion:**

A significant physical fatigability could be shown in euthyroid HT patients despite missing obvious neuromuscular deficits in routine testing. Further, elevated pain and fatigue perception in HT patients seem to contribute to nonspecific muscle complaints in these patients. A possible pathogenic role of thyroid autoimmunity in hidden neuromuscular involvement may be suggested.

## Introduction

It is well known that neuromuscular symptoms can be associated with thyroid dysfunction. Here, muscle weakness is observed more in hypothyroidism than in hyperthyroidism, exhibiting clinical features that suggest functional myopathy [9]. Hashimoto thyroiditis (HT) is the most prevalent autoimmune disease worldwide [12]. It is a T cell-mediated disease characterized by elevated levels of serum anti-thyroidperoxidase (anti-TPO) antibodies (ab) and shows a typical sonographic pattern. HT mostly leads to hypothyroidism. Neuromuscular symptoms in hypothyroidism alone are reported in 30–80% of patients, and these symptoms usually improve or disappear when the hypothyroid state is corrected [12]. However, some symptoms of HT, such as profound fatigue, poor sleep quality, and muscle pain, frequently persist as an interfering symptom burden not related to hypothyroidism, but rather to the autoimmune disease itself [12, 14, 27]. Total thyroidectomy seemed to improve health-related quality of life and fatigue in these patients [12].

Prospective data on neuromuscular involvement in HT patients are rare. The aim of this study therefore was to investigate the prevalence of neuromuscular symptoms and to assess objective motor fatigability in euthyroid HT patients beyond electrophysiological findings, muscle pain, and subjective fatigue.

## Methods

### Patients/sample

A total of 24 HT patients (23 women, 1 man) aged 21 to 69 years [mean (M) = 39.0, standard deviation (SD) = 12.6] and 25 healthy controls (23 women, 2 men) aged 23–64 years (*M* = 36.1, SD = 13.5) were prospectively included in the study (Table 1). Age and gender did not differ in the two groups. Only patients in whom the thyroid gland had not been resected were included in the study.

**Table 1:** Characteristics of patients and controls

	Hashimoto patients	Controls
Number of participants, m:f	24; 1:23	25; 2:23
Age, years, mean (SD, SE)	39.0 (12.6, 2.6)	36.1 (13.5, 2.7)
BMI, mean, (SD, SE)	25.07 (4.8; 1.0)	23.01 (3.0; 0.6)
BMI < 25/25–30/ > 30–35	48%/35%/17%	68%/32%/0
Duration of illness, months, mean (SD,SE)	73.3 (58.2, 11.9)	
Smoker	7 (29.2%)	5 (20%)
**Lab analysis**		
Anti TPO ab above 35 IU/ml at examination	18/24 (75%)	
Anti-TPO range (IU/ml)	38–600 (median 211)	

None of the parameters were significantly different in patients and controls

*SD* standard deviation, *SE* standard error of the mean

The diagnosis of HT was based on positive testing of serum anti-TPO ab and a typical inflammatory pattern in thyroid ultrasound. Values of current anti-TPO ab are shown in Table 1.

All HT patients were on thyroid replacement therapy and thyroid gland function had been normal for at least 6 months at the time of the examination. According to the protocol, no patient was taking steroids (for clinical details, see Table 1) or suffered from a manifest neuromuscular disease. No pathologic decrement in repetitive nerve stimulation (RNS) of the accessory nerve was observed in any patient before physical testing. Patients and controls with other neurological disease or relevant cardiac, pulmonary, and orthopedic limitations that might potentially have a significant impact on current exercise tolerance, such as sleep apnea syndrome or limiting mobility, were excluded from the study. Other exclusion criteria were history of drug or alcohol abuse, psychiatric illnesses, or considerable limitations in hearing or vision.

The study was approved by the local ethics committee of the Medical Faculty of the University Halle-Wittenberg. Written informed consent was obtained from all participants before study inclusion.

### Clinical examination

After inclusion in the study, all patients were carefully clinically examined. In all patients, rheumatic disease was excluded according to the American Rheumatic Association criteria [1]. Sensory and motor function, including paresis (including functional testing of squats and hop ons), walking characteristics, and sensory and coordination skills, were carefully documented by the same neurologist (Table 2). Patients were questioned for clinical symptoms of neuropathy, such as dysesthesia, burning sensations, muscle cramps, myalgia, weakness, and arthralgia. Dynamometer testing with a hydraulic hand dynamometer (^©^2008, Rehaforum MEDICAL) was used to quantify maximal strength (in pounds) in isometric contraction of the hand (determined in 3 runs).

**Table 2:** Clinical and neurophysiological characteristics of patients

	Hashimoto patients, *n* = 24
**Muscular symptoms (patient reported)**	
A Exercise-induced weakness	13/24
B Cramps	7/24
C Myalgia	5/24
2 out of A/B/C	5/24
3 out of A/B/C	2/24
**Sensory neuropathy**	
Paresthesia	1/24
Numbness including reduced vibration sense	4/24
Small fibre symptoms*	None
Sensory ataxia	1/24
Autonomic neuropathy	1 (Hyperhidrosis)
Decreased ankle jerk	1/24
Weakness or foot muscle atrophy	0/24
**Neurophysiology**	
Pathologic decrement in 3 Hz stimulation	0/24
Motor neuropathy (measuring NCV of tibial nerve)	0/24
Sensory neuropathy (measuring NCV of sural nerve)	2/24 (1 mild axonal, 1 moderate axonal)
Configuration of MUAPs**	
M. vastus lateralis	24/24 normal
M. tibialis anterior	23/24 normal, 1/24 mild neurogenic
Paravertebral thoracic 10	23/23 normal
Increased insertional activity	3/24 (2 M. tib. ant, 1 thoracic paravertebral)
Positive sharp waves	2/24 (1 M. vast. lat., 1 thoracic paravertebral)

*SD* standard deviation, *SE* standard error of the mean

^*^Intolerance of temperature and touch, burning

^**^Motor unit action potential

In addition, nerve conduction velocities (NCV) of sural and tibial nerves, including F-waves, were measured. A NCV of at least 40 m/s and amplitudes of at least 5 mV (tibial nerve) and 5 µV (sural nerve) were deemed as normal. An electromyography (EMG) of vastus, tibial, and paravertebral muscles of 10th thoracal level was performed.

### Physical testing methods

#### Arm movement test (AMT) [15]

In a sitting position, subjects were asked to hold a standardized item weighing 500 g (filled water bottle, easily manageable for all participants) with the arm horizontally outstretched and to move it repeatedly between 2 points, forming a horizontal quarter circle as rapidly as possible. Within 90 s of testing, the number of correct bottle placements (taps) was assessed in 6 subintervals (15 s each). The mean number of taps per subinterval was indicated as individual motor performance level in AMT. RNS of accessory nerve was performed directly within 1 min before and after finishing the test to unmask any neuromuscular transmission defect [18].

#### 6-min-walk-test (6MWT)

Performance of 6MWT followed a standard protocol [15]. Participants were asked to walk for 6 min on a hard, flat surface as fast as they were able to. Distance (in meters) traveled per minute was measured. This gave 6 consecutive results of subintervals. The mean distance achieved per subinterval represented the individual motor performance level on the 6MWT.

### Questionnaire assessments

Additionally, HT patients and control subjects completed the following validated questionnaires:

Fatigue Scale for Motor and Cognitive Function (FSMC) [20]. This 20-item, comprehensive, self-report questionnaire assesses cognitive and physical fatigue in two subscales (maximum value per subscale: 50) in MS patients. For the subscale of cognitive and physical fatigue, scores above 22 were defined as “mild”, above 28 as “moderate”, and above 34 as “severe” fatigue [20].

Pittsburgh Sleep Quality Index (PSQI), an 18-item assessment of subjective sleep quality during the preceding 4 weeks [5].

Center for Epidemiological Studies Depression Scale-Short Form (CES-D-SF) [24], an assessment of mood, somatic complaints, and motor inhibition during the last week.

PSQI and CES-D were applied to unmask confounder effects of sleep disturbances on physical performance and perception of fatigue which could be ruled out in regression analysis.

#### Pain scores

Brief pain inventory (BPI) was used to assess pain in both groups [8]. The pain severity score includes different pain levels, including current pain. The pain interference score is based on social life, mood, sleep, walking, and working activities [8].

#### Test sequence

AMT and 6MWT were run after the clinical neurological examination. Before and after completing the tests, participants assessed their current, perceived physical and cognitive performance level using a 10-point Visual Analog Scale (VAS) ranging from one (very low) to 10 (very high).

#### Data analysis

Linear trend (LT) was calculated to measure physical fatigability as the dynamic in AMT and 6MWT performance, using the subsequently recorded number of taps (bottle placements in AMT) and distance achieved (6MWT) within fixed 15-s and 60-s time intervals, respectively [4, 15, 25].

The LT for the 6MWT and AMT was calculated according to the formula below [4, 15]. The result “R” (including R1 to R6) represents the distance reached in each of 6 intervals in walking (6MWT) and, accordingly, the bottle taps in 6 time intervals (AMT).

$${\text{LT}}_{{\text{6MWT/AMT}}} = \, \frac{{\left[ {\left( { - 5 \times {\text{R}}1} \right) + \left( { - 3 \times {\text{R}}2} \right) + \left( { - 1 \times {\text{R}}3} \right) + \left( {1 \times {\text{R}}4} \right) + \left( {3 \times {\text{R}}5} \right) + \left( {5 \times {\text{R}}6} \right)} \right]}}{70}$$

Thus, LT was applied to reflect the degree of deviation from a constant course of performance. An LT of 0 describes a stable performance; an LT < 0 indicates a decline in performance and is a correlate of physical fatigability. The LT is independent of the absolute level of performance in accordance with the formula.

A *t* test for unpaired samples was run to compare results of HT patients and the control group in the physical testing parameters and the questionnaire scores (Table 3). In cases of variance inhomogeneity, the Mann–Whitney–Wilcoxon *U* Test was appropriately applied as an alternative nonparametric testing method. Testing was evaluated using IBM SPSS Statistics Version 23.0.

**Table 3:** Physical testing procedures and assessment scores

	Hashimoto patients (M ± SD)	Controls (M ± SD)
Number of participants	24	25
**Physical fatigue assessment**		
*Dynamometer*		
Grip [pounds]	61.1 ± 18.6	63.4 ± 10.6
*Arm movement test (AMT)*		
Number of taps/15 s (Motor performance level)	28.2 ± 7.03	29.1 ± 8.5
Linear trend	0.11 ± 0.46	0.12 ± 0.64
*6-min walk test (6MWT)*		
Distance /60 s, m (Motor performance level)	92.15 ± 16.7	98.9 ± 13.1
Linear trend (LT)	− 0.93 ± 1.79*	− 0.01 ± 0.78
**Questionnaire scores**		
* Pain perception*		
Pain severity score	2.2 ± 2.48**	0.23 ± 0.6
Pain interference score	2.3 ± 2.73**	0.12 ± 0.39
*Physical performance level*		
VAS before testing (range 0–10)	6.6 ± 1.9*	7.8 ± 1.6
VAS after testing (range 0–10)	6.2 ± 2.3**	7.8 ± 1.7
*Cognitive performance level*		
VAS before testing (range 0–10)	7.0 ± 1.8	7.8 ± 1.4
VAS after testing (range 0–10)	7.2 ± 1.9	7.9 ± 1.4
* Others*		
CES-D-SF (range 0–45)	13.7 ± 7.5*	8.9 ± 5.1
PSQI (range 0–21)	7.6 ± 3.4**	4.3 ± 2.9
FSMC total (range 20–100)	61.4 ± 20.7**	31.7 ± 9.6
FSMC cognitive (range 10–50)	31.0 ± 11.2**	15.8 ± 4.6
FSMC physical (range 10–50)	30.4 ± 10.3**	16.9 ± 7.4

*AMT* arm movement test [15],* 6MWT* 6 min walk test [15], *M* mean, * SD* standard deviation,* CES-D-SF* Center for Epidemiological Studies Depression Scale-Short Form [24], *PSQ* Pittsburgh Sleep Quality Index [5], *VAS* visual analog scale (Subjective rating of physical performance level), *FSMC* fatigue scale for motor and cognitive function [20]

^**^p < 0.005, *p < 0.05 (*p* value; unpaired *t* test, 2-sided, comparing Hashimoto patients with controls)

## Results

### Neurological assessment and neurophysiological findings

At clinical examination, no weakness of the extremities was discovered in HT patients. The most dominant finding was sensory neuropathy (especially on feet), which could only be demonstrated neurophysiologically in 2 of the 24 patients. EMG revealed increased insertional activity in 3 of the 24 patients and positive sharp waves in 2 of the 24 patients. Muscle action potential analysis and recruitment pattern did not reveal any abnormalities consistent with myopathy. All clinical and neurophysiological findings are summarized in Table 2.

### Blood tests

Creatine kinase (CK), myoglobin, and thyroid hormone (FT3, TSH) levels were normal.

All HT patients were negative for ab against titin, muscle specific tyrosine kinase (MuSK) receptor, low-density lipoprotein receptor-related protein 4 (LRP4), aquaporin, glutamic acid decarboxylase (GAD), voltage-gated potassium channels, acetylcholine receptor (AChr) ab, and antinuclear ab.

### Objective measurement of physical fatigability

**Fig. 1:**
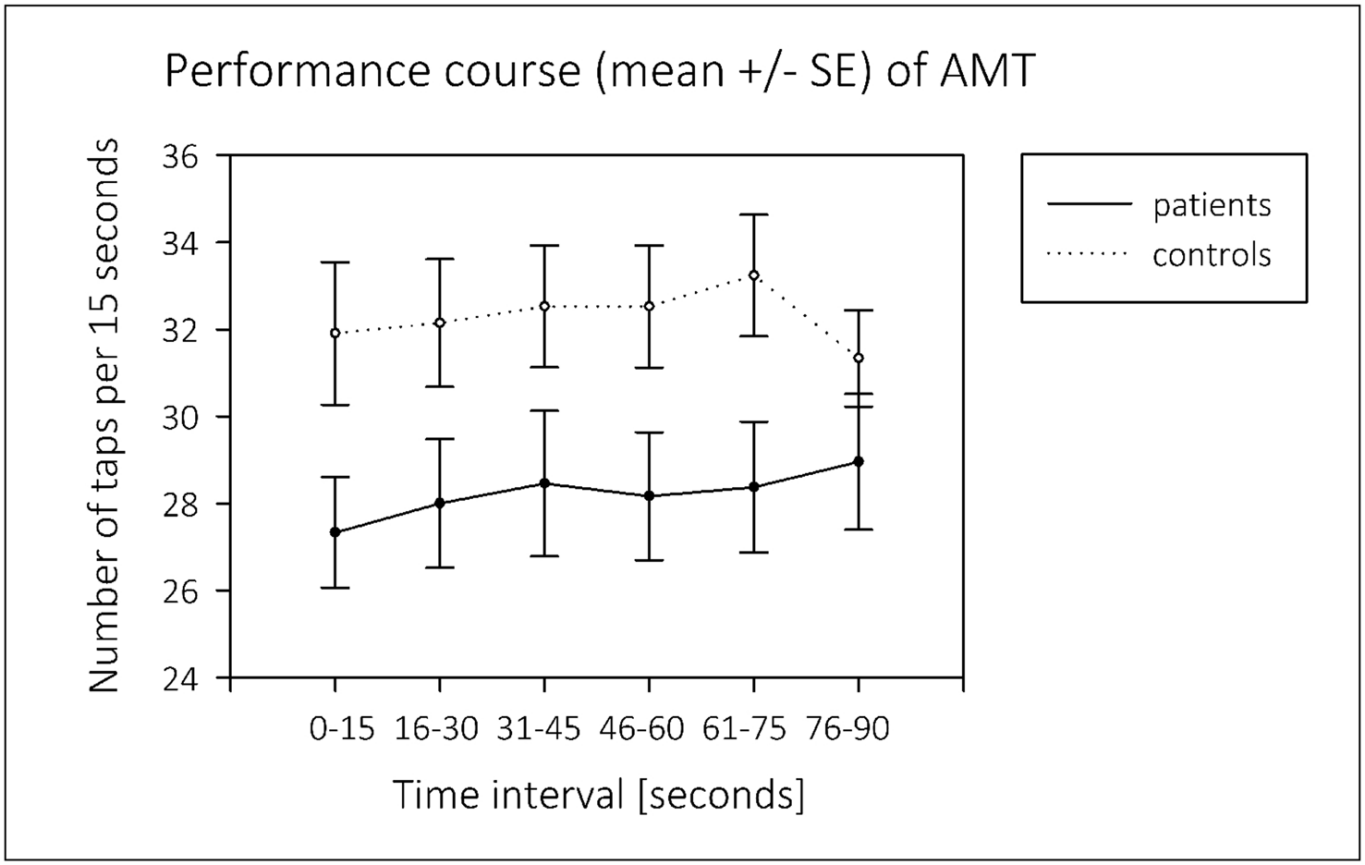
Linear trend in AMT. AMT arm movement test, SE standard error

There was no difference between groups in dynamometer grip and motor performance levels in both AMT and 6MWT (Table 3). The mean value of the LT in AMT was positive and did not differ between groups (Fig. 1).

**Fig. 2:**
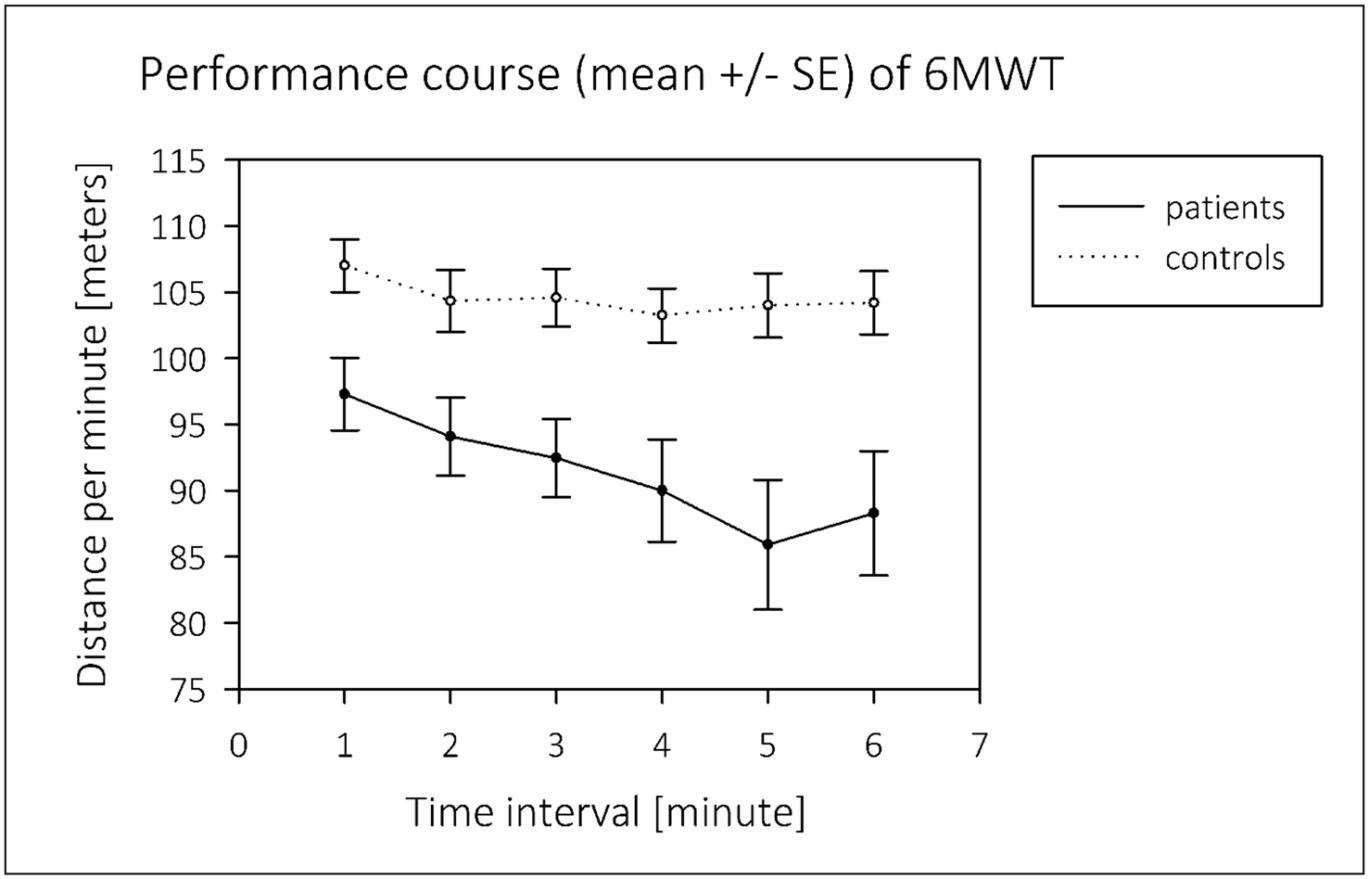
Linear trend in 6MWT. 6MWT 6 min walk test, SE standard error

In 6MWT, LT in HT patients was negative and significantly differed from the control group, indicating a gradual decrease in walking performance as an objective parameter of fatigability (Table 3, Fig. 2). However, control subjects also presented with a slightly negative LT in 6MWT (Table 3). In HT patients, LT correlated with motor performance level in 6MWT (Pearson correlation coefficient *r* = 0.69, *p* < 0.001) but not in AMT. This means that cumulative walking distances were longer in patients with positive LT. An influence of mood (CES-D-SF) and sleep quality (PSQI) as well as pain, ab titer, and Body Mass Index (BMI) on LT could be excluded in regression analysis.

### Questionnaires: fatigue, pain, sleep, and mood

A significant difference was evident between HT patients and controls regarding sleep quality (PSQI) (*p* < 0.001) and CES-D-SF assessment (*p* < 0.05) (Table 3).

Self-assessment of the FSMC scores revealed significantly higher fatigue levels in HT patients than in controls as well as increased pain perception (Table 3). Based on FSMC score, 18/25 (72%) control subjects did not experience physical fatigue, whereas in the HT group, 3/24 (7.5%) patients presented with mild, 4/24 (17%) with moderate, and 8/24 (33%) with severe physical fatigue.

In regression analysis, the physical FSMC score was primarily influenced by pain perception (standardized regression coefficient beta = 0.633, *p* = 0.002), whereas an effect of all other correlating factors (PSQI r = 0.61**, CES-D-SF *r* = 0.52**, pain interference score r = 0.55**) could be ruled out in regression analysis.

Pain severity scores moderately correlated with BMI (*r* = 0.64**, beta = 0.3, *p* = 0.001) and PSQI (r = 0.49*, beta = 0.341, *p* = 0.039) and the pain interference score also correlated with BMI (*r* = 0.546**, beta = 0.397, *p* = 0.001) and FSMC cumulative score (*r* = 0.722**, beta = 0.561, *p* = 0.039).

Neither pain score nor physical fatigue scores showed a correlation with 6MWT-LT nor did anti-TPO ab titer, disease duration, or CES-D-SF score.

## Discussion

### Neuromuscular findings in HT including neurophysiology

Clinical experience treating patients with chronic lymphocytic thyroiditis (HT) suggests that common neuromuscular symptoms develop in these patients despite long-standing euthyroidism [2]. In our study, 13/24 (54%) patients reported physical exhaustion in everyday life. Seven/24 (29%) suffered from cramps, and 5/24 (21%) from myalgia. This is in line with the results of Villar et al. [28], showing that 9/10 HT patients reported muscular cramps and 7/10 of HT patients distal weakness. Proximal weakness was not observed. Giuffrida reported that 28/62 HT patients with a normal thyroid function suffered from myalgia [11].

In the early course of thyroid diseases, sensorimotor axonal neuropathy has been reported in 40% of patients with hypothyroidism and 20% with hyperthyroidism [9], whereas El-Salem described demyelinating neuropathy in 52% of asymptomatic patients with hypothyroidism [10]. These findings, together with muscle weakness in 38% of patients with hypo- and 62% with hyperthyroidism, respectively, are primarily explained by metabolic dysfunction [9]. Data on neuropathy (not further specified) in euthyroid HT patients are rare and that study showed a prevalence of 6/53 cases (11.1%) [2]. Indeed, Bai postulated that neuropathy was neither closely correlated to thyroid function nor to ab titer. In our series, sensory neuropathy was clinically evident in 4/24 (16.7%) and could be reproduced as sural axonal damage in NCV in 2 patients, which might indicate that the fibers were mildly affected in a certain way. Consistent with Villar [28], NCV was normal in all patients. However, in a 52-year-old woman, motor neuropathy was found only by neurogenic firing in distal leg muscles in EMG and correlated with reduced ankle jerk. We agree with Bai [2] that neuropathy occurs more often in patients with chronic HT. Our patients affected by neuropathy were aged between 45 and 69 years, suggesting that persisting autoimmune dysfunction might predispose to neuropathy.

In addition, the presence of myopathy has been suspected in HT according to previous case series that included histological data [2, 17, 26, 28]. Here, focal muscle inflammation, common in autoimmune diseases, could be demonstrated in 10/10 HT patients [28]. Histological changes also included moderate-to-severe atrophy, necrosis, and muscle capillary alterations, increased proportion of type II fibers, and positive reactions for immunoglobulin in 80% of patients [17, 28]. Necrosis was not sufficiently widespread to increase CK levels [28]. In HT, augmented metabolic effects due to endocrine dysfunction are thought to affect muscle pathology, but autoimmune processes might directly induce myopathy, too [10]. However, in the series by El-Salem, the intensity of clinical symptoms was not related to muscle findings, the EMG results, or to the functional state of the thyroid gland [28].

In our patients, there were no myogenic changes in EMG, but in 5/24 either positive sharp waves or increased insertional activity was found, which might be indicative for some mild myopathy (Table 2). This is in contrast to Villar’s data, demonstrating normal EMG (without abnormal spontaneous activity) at rest but low amplitudes and short-duration polyphasic motor unit potentials in 5/10 patients during contractions (more frequently found in deltoid and quadriceps femoris muscles). These data and the aforementioned histological findings should be interpreted with caution, however, as in 5/10 patients, thyroid function was subclinical or overtly deficient due to HT [28].

Especially pathologic spontaneous activity in paravertebral muscles might suggest existing myopathy [21]. In our study, only 2 patients presented slight paravertebral abnormalities (Table 2). However, we argue that histological muscle pathology in HT is suspected to be focalized and therefore may explain different findings in EMG as well [28]. Elevated CK may be apparent owing to thyroid hormone disorders not correlating with weakness or hormone levels [9]. None of our patients showed an elevated CK level.

### Autoimmune pattern in HT patients

Autoimmune patterns in thyroid diseases are well known and could be diagnosed in 92 (26.8%) of myasthenia gravis (MG) patients, including in 4.4% with Graves’ disease, 9% with HT, and 13.4% with antithyroid ab only [16]. MG coexisting with autoimmune thyroid diseases follows a milder course than MG alone. On the other hand, elevated AChR ab were found in 13/53 (24.5%) HT patients [2]. This might at least partially explain the high rate of 13/29 (44.8%) patients with neuromuscular disorders in this cohort. In our patients, no ab predisposing to MG or other autoimmune disease except for thyroid disease was found.

### Objective fatigability in HT patients

Fatigability can be determined by applying the LT method as we previously showed in MG patients [15]. Based on this method, we revealed a significant physical fatigability in HT patients compared to controls in 6MWT. Fatigability from a repetitive arm movement task in HT was not evident.

Consistent with data in MG patients, HT patients with a higher motor walking level showed a reduced fatigability in 6MWT (in regression analysis correlation of motor performance level with LT in 6MWT beta = 0.681, *p* = 0.001)[15]. However, in HT patients, the performance level in 6MWT declined more impressively (LT mean − 0.93 ± 1.79 SD) than in MG patients (LT mean − 0.62 ± 1.43) [15]. This is remarkable as HT patients were younger and their performance level was higher than for MG patients (mean 92.15 ± 16.7 versus 61.6 ± 21.8, respectively) [15].

Based on regression analysis, neither disease duration nor ab titre, pain scores, BMI, mood, or sleep quality scores nor subjective fatigue perception (FSMC) affected objective fatigability. Therefore, we suggest that fatigability in 6MWT seems to be a suitable independent and objective parameter of neuromuscular involvement in HT. The cause of fatigability is not well understood yet. We argue that it might be explained by coexisting hidden autoimmune inflammatory mechanisms and metabolic dysfunction, especially in proximal muscle groups, which contribute to neuromuscular involvement in Hashimoto patients.

### Association of pain and subjective fatigue in HT patients

Patients with HT often report profound fatigue, but also poor sleep quality and both muscle and joint tenderness. However, these symptoms sometimes consistently persist despite a euthyroid status while receiving hormone substitution [6, 12, 14, 19, 27]. Consistent with these data, we found that pain and fatigue perception is significantly increased in HT patients compared to controls. Here, 50% of HT patients reported proximal muscle pain, 25% other neck pain involvement. Pain was lancing in 5/12, burning in 4/12, and dull in 6/12 patients. This is consistent with a previous study reporting on 6/10 patients with muscle pain associated with cold [28] and fatigue ranging from 57 to 82% [12, 28]. Persistence of pain and fatigue as possible signs of muscle disturbances in HT patients despite long-standing, adequate thyroid hormone replacement is somewhat obscure. One hypothesis holds that complete muscle recovery takes a long time after hormonal levels normalize. However, chronic autoimmune mechanisms, and not subclinical hypothyroidism alone, are postulated to predispose to muscle pain (including pain and fatigue in VAS) in HT patients [3, 7, 11, 22]. Consistent with this, fatigue declined significantly from 82 to 35% by thyroid surgery, correlating also with decline in ab and improvement in health-related quality of life (SF 36) [12, 23].

In our study, pain perception seems to moderately correlate with BMI and PSQI scores but not with objective fatigability (LT), anti-TPO ab titer, or mood. Mood and sleep quality in HT patients were significantly deteriorated in comparison to controls. However, an influence of mood and sleep on objective physical fatigability measurements or on physical FSMC was not found.

In our patients, pain perception did strongly correlate with physical FSMC score in regression analysis (beta = 0.633**). This emphasizes the well-known impact of musculoskeletal pain on the biopsychosocial perspective, including its downstream effects on contextual factors, such as sleep interruption, fatigue, depressed mood, activity limitations, and participation restrictions [13]. This should direct our attention to more frequent, nonspecific complaints in women with HT in the absence of other systemic comorbidities and regardless of thyroid functional status.

## Conclusion

In our study, a significant physical fatigability could be shown in euthyroid HT patients. Fatigability may develop despite normal clinical, neurophysiological, and laboratory criteria. Furthermore, pain and fatigue were significantly elevated in HT patients and correlated with each other. Our findings strengthen the theory of a possible pathogenic role of thyroid autoimmunity in hidden neuromuscular involvement and nonspecific muscle complaints in these patients. Therefore, conducting LT testing in 6MWT to evaluate walking fatigability might constitute a valuable additional diagnostic tool. Increased attention to pain and fatigue assessment may help to manage symptoms better in HT patients.

## Data Availability

Data available from the authors upon request.
